# Comparative Effectiveness of Smoking Cessation Medications: A National Prospective Cohort From Taiwan

**DOI:** 10.1371/journal.pone.0166992

**Published:** 2016-11-28

**Authors:** Po-Yin Chang, Po-Ching Lo, Hui-Chin Chang, Kuang-Chieh Hsueh, Yi-Wen Tsai

**Affiliations:** 1 Department of Medicine, Stanford University School of Medicine, Stanford, California, United States of America; 2 Institute of Health and Welfare Policy, National Yang-Ming University, Taipei, Taiwan; 3 Kaohsiung Veterans General Hospital, Kaohsiung, Taiwan; Centre for Addiction and Mental Health, CANADA

## Abstract

**Background and objective:**

Relative effectiveness of smoking cessation medications—varenicline, bupropion and nicotine replacement therapy (NRT)—remains unclear among smokers in real-world settings. Evidence in females and smokers with light/moderate nicotine dependence is particularly insufficient. This study compared the effectiveness of varenicline, bupropion or NRT gum relative to NRT patch, in achieving abstinence among recent quitters.

**Methods:**

In a national smoking cessation program in Taiwan (2012–2015), a cohort of 11,968 participants received varenicline (n = 5,052), bupropion (n = 823), NRT gum (n = 1944) or NRT patch (n = 4,149). The 7-day, 1-month or 6-month point-prevalence was calculated based on self-reported last smoking event via telephone interview after 6 months. Logistic regression modellings estimated odds ratios (OR) and 95% confidence intervals (CI) for achieving abstinence using different modalities (NRT patch as referent). Models included age, sex, education, marital status, geographic region, smoke-years, nicotine-dependence level, medical institution, number of clinic visits and medication use duration. Analyses were further stratified by sex and dependence severity.

**Results:**

Participants were predominantly male (83%) with a mean age of 43.7±12.6 years. Varenicline users were more likely than NRT patch users to achieve abstinence, based on 7-day point-prevalence (OR = 1.30, CI: 1.19–1.44), 1-month point-prevalence (OR = 1.36, CI: 1.24–1.50) or 6-month point-prevalence (OR = 1.30, CI: 1.14–1.47). Compared with NRT patch, varenicline was associated with greater odds of being abstinent in women (OR = 1.29, CI: 1.01–1.65), men (OR = 1.31, CI: 1.18–1.46), those with light/moderate dependence (OR = 1.42, CI: 1.24–1.63) or smokers with severe dependence (OR = 1.19, CI: 1.04–1.37), based on 7-day point-prevalence. Differences in effectiveness were not observed between users of bupropion, NRT gum and NRT patch.

**Conclusions:**

In smoking cessation clinics in Taiwan, varenicline users reported higher abstinence rates than NRT patch users after 6 months. Women and smokers with light/moderate nicotine dependence may also benefit from varenicline in actual clinical practice.

## Introduction

Tobacco smoking inflicts substantial ill-health, shortens life-expectancy by 10 years or more, and increases mortality [1, 2]; quitting before age 55 can gain smokers 6 to 10 years of life compared with others who continue to smoke [2]. Nicotine replacement therapy (NRT), bupropion and varenicline are common medications used individually or in combination to achieve smoking cessation and have been recommended by the United States (US) Preventive Services Task Force [3].

Varenicline, an α4β2 nicotinic acetylcholine receptor partial agonist, was introduced to worldwide markets in 2006. Individual clinical trials and meta-analyses suggested that varenicline was more effective than single-use NRT (patch, gum, or other formulations) [3–5], or bupropion [5–7]; whereas bupropion had similar efficacy to NRT [3, 4]. Most studies comparing the effectiveness of smoking cessation medications were clinical trials in the US [3], and there has been a limited number of studies comparing varenicline to NRT in actual clinical practice [8–10]. Moderate sample sizes [8–10], cohorts from a single health care system [8] and cross-sectional study design [9] may restrict their relevance beyond these settings. The EAGLES study was the first clinical trial comparing relative neuropsychiatric safety and efficacy of varenicline and bupropion with NRT (active-control group) and placebo group in the US, Europe, and South and Central America [11]. Varenicline showed greater efficacy than bupropion or NRT, and there was no difference in efficacy between bupropion and NRT [11].

The effectiveness of smoking cessation medications may vary according to users’ sex and nicotine dependence severity. Female smokers have more difficulty in quitting and may respond differently than males to smoking cessation medications [12, 13]. Although some studies observed no differences between males and females in the efficacy of NRT patch, bupropion or varenicline [7, 13–15], one recent analysis reported greater efficacy of NRT patch in men [16]. Smoking behaviors have changed too. For example, compared to smokers in 2005, those in 2011 smoked fewer cigarettes per day [17]. However, results from a randomized controlled trial suggested that greater effectiveness of varenicline versus NRT may not be evident among less nicotine-dependent smokers or light smokers [18]. In clinical trials, participants received intense monitoring, which may differ from a ‘real-world’ situation. The relative effectiveness of varenicline compared to NRT in general Asian populations has remained uncertain. Few studies in real-world settings have examined the comparative effectiveness of different smoking cessation medications in men versus women, or in smokers with differing levels of nicotine dependence.

The primary objectives of this large prospective cohort study were to use the national smoking cessation program in Taiwan to: 1) examine the comparative effectiveness of NRT patch or gum, bupropion or varenicline in achieving short- and intermediate-term abstinence; 2) investigate the relative effectiveness of four smoking cessation modalities by sex and, separately, by nicotine dependence. We found varenicline, but not bupropion, to be superior to NRT patch in achieving self-reported abstinence among recent quitters, including both females and lightly/moderately nicotine-dependent smokers.

## Materials and Methods

### Study population

The Health Promotion Administration in Taiwan began the Second Generation Tobacco Smoking Cessation Services Program on 1 March 2012. This program provided subsidized co-payments for smoking cessation medications. Adults aged 18 years or older seeking to quit tobacco smoking were eligible to receive a single smoking cessation pharmacotherapy and/or health education at smoking cessation clinics, with up to two treatment courses per year. In one treatment course, participants could obtain ≤8 prescription refills (a 90-day supply) for the same smoking cessation medication from the same clinic. Smoking cessation medications included NRT (patch, gum, inhaler or tablet), bupropion (tablet), or varenicline (tablet). Pregnant women or individuals younger than 18 were eligible for health education. At their first clinic visit, participants provided demographic information (age, sex, marital status, education, etc.) and were prescribed smoking cessation medications with an evaluation for smoking-related behaviors including the Fagerström Test for Nicotine Dependence (FTND) [19]. During follow-up, smoking reduction, side effects from medications, nicotine withdrawal syndrome, and changes in prescriptions were reported at approximately 3-monthly intervals. Each month, a sample of 1,000–4,000 participants was randomly selected to participate in a telephone interview 6 months after their first visit, to assess their smoking cessation status. Data were collected and analyzed from 11,968 participants who successfully completed the interview.

### Ethics statement

The National Yang Ming University Institutional Research Board, Taipei, Taiwan, approved the study protocol (YM104027E). The Board waived informed consent of participants because they were not individually identifiable from the data analyzed.

### Smoking cessation medications

This study targeted smokers prescribed monotherapy with NRT, varenicline or bupropion in their first treatment course. Smoking cessation medications subsidized by the Tobacco Smoking Cessation Services Program included six NRT patch products (including both brand-name and generic products, dosage per patch ranged from 10.4 to 52.5 mg), six NRT gum products (brand-name products only, dosage per piece ranged from 2 to 4 mg), eight bupropion products (including both brand-name and generic products, dosage was 150 mg), and two varenicline products (brand-name products with dosage 0.5 mg or 1.0 mg). Participants were categorized into four groups: NRT patch, NRT gum, varenicline or bupropion use in the first treatment course. We focused on monotherapy among first-time users to avoid the mixed-effects of combined or add-on drugs. Medications in the second treatment course were excluded to prevent the carry-over effects from the previous treatment.

### Point-prevalence of abstinence after 6 months

Three smoking cessation rates at the 6-month telephone interview were derived from participants’ responses to the question: “When was the last time you smoked? < 1 day, 1–6 days ago, 7–29 days ago, 30–179 days ago or 180 days ago?” The 7-day, 1-month and 6-month point-prevalence of abstinence indicated the proportion of participants who self-reported last smoking 7 days ago or more (“7–29 days ago”, “30–179 days” or “180 days ago”), at least 30 days ago (“30–179 days ago” or “180 days ago”) and 6 months ago (“180 days ago”), respectively, during the phone interview.

### Statistical analysis

Baseline characteristics were presented in mean (± standard deviation) for continuous variables or number (percentage) for categorical variables, and compared by one-way ANOVA (continuous variables) or chi-square test (categorical variables) across the four participant groups. Multivariable logistic regression models estimated odds ratios (ORs) and 95% confidence intervals (CIs) for 7-day point-prevalence of abstinence, comparing three smoking cessation modalities against NRT patch as the reference. Multivariable regression comparing four treatment groups (NRT patch reference) was also performed to estimate the ORs for 1-month point-prevalence of abstinence and, separately 6-month point-prevalence. Models included age (per 10-year change in age), sex (male/female), education level (unknown/none/elementary, junior high school, senior high school or college or higher), marital status (single, married or other), geographic region (North, West-central or South Taiwan), continuous smoke-years (per 10-year change), nicotine dependence level (light or moderate vs. severe nicotine dependence), medical institution (community clinic or hospital outpatient clinic), number of clinic visits and continuous prescription duration (in weeks). Nicotine-dependence level was stratified according to the FTND score: light (score 0–3), moderate (score 4–6), or severe (score 7–10). The continuous medication use duration, which was the sum of the total days of refilled prescriptions at each clinic visit during the first treatment course, was used as a proxy for the potential dose-effect.

To explore whether smoking cessation medications have differential effectiveness on abstinence in clinical practice, we also performed multivariable regression analyses stratified by dependence severity (light or moderate vs. severe) and separately by sex, as suggested in clinical trials [3, 18, 20].

**Fig 1:**
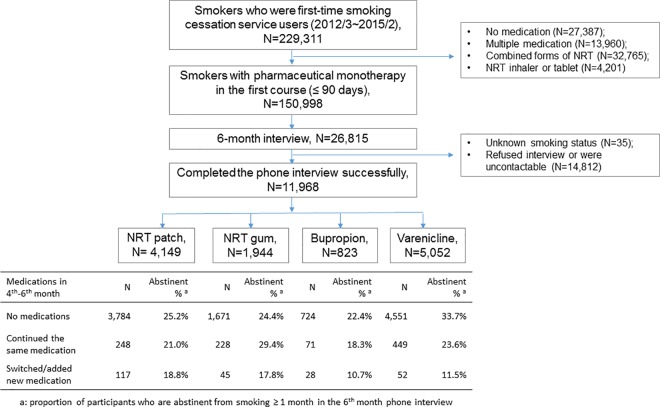
Participant selection and abstinence outcomes. NRT, nicotine replacement therapy.

Before the telephone interview, a small proportion (4.6%) of participants had switched to or added new smoking cessation medications in the second treatment course (4–6 months after the first clinic visit) (Fig 1). We included these participants in the sensitivity analysis and defined their outcome as being smokers because the new medications suggested the smoking cessation aids in the first treatment course were not sufficient for achieving abstinence. In a separate sensitivity analysis, we included 14,847 participants who did not complete the telephone interview or whose smoking status was not established.

All *P*-values were two-sided, with *P* < 0.05 considered statistically significant. All statistical analyses were performed using STATA software (version 11.1; Stata, College Station, TX, USA).

## Results

From March 1, 2012, until February 28, 2015, 150,998 adult smokers (age ≥18 years) were prescribed one type of medication during their first visit for smoking cessation (Fig 1). A random sample of 26,815 was scheduled to receive a follow-up telephone interview 6 months later, of whom 14,847 were excluded for analyses: 14,812 (55%) refused the interview or could not be reached and another 35 had unknown smoking status. The sample population consisted of 11,968 adult smokers who successfully completed the interview.

### Cohort characteristics

The study cohort consisted of predominantly male smokers (80%) with a mean age of 43.7 years; 63% to 67% in the four treatment groups were aged 35 to 64 years (Table 1). Among these 11,968 first-time users of single medications for smoking cessation, varenicline was prescribed most often (42%), followed by NRT patch (35%), NRT gum (16%) and bupropion (7%). Men were more likely to have received NRT or varenicline than bupropion (*P* < 0.001).

**Table 1:** Demographic characteristics of participants by medication type

Variables: data show number (%) or mean ± SD	NRT patch	NRT gum	Bupropion	Varenicline	*P*-value^a^
	(*n* = 4,149)	(*n* = 1,944)	(*n* = 823)	(*n* = 5,052)	
**Age, year**					
Mean ± SD	44.6 ± 11.9	43.8 ± 13.3	41.4 ± 11.5	43.3 ± 12.1	
18–24	209 (5)	143 (7)	37 (5)	233 (5)	<0.001
25–34	874 (21)	436 (22)	235 (29)	1189 (24)	
35–44	1166 (28)	485 (25)	261 (32)	1524 (30)	
45–54	1025 (25)	481 (25)	180 (22)	1207 (24)	
55–64	574 (14)	270 (14)	79 (10)	675 (13)	
≥65	301 (7)	129 (7)	31 (4)	224 (4)	
**Male (participants)**	3452 (83)	1676 (86)	665 (81)	4292 (85)	<0.001
**Education level**					<0.001
None, elementary, or unknown	503 (12)	202 (10)	58 (7)	384 (8)	
Junior high school	561 (14)	300 (15)	125 (15)	604 (12)	
Senior high school	1660 (40)	754 (39)	330 (40)	1859 (37)	
Undergraduate or higher	1425 (34)	688 (35)	310 (38)	2205 (44)	
**Marital status**					0.001
Single	1009 (24)	517 (27)	239 (29)	1347 (27)	
Married	2578 (62)	1176 (60)	485 (59)	3142 (62)	
Other	562 (14)	251 (13)	99 (12)	563 (11)	
**Geographic area**					<0.001
North	1877 (45)	750 (39)	351 (43)	2790 (55)	
West-central	1066 (26)	570 (29)	235 (29)	900 (18)	
South	1206 (29)	624 (32)	237 (29)	1362 (27)	
**Medical institution**					<0.001
Community clinics	3009 (73)	1589 (82)	765 (93)	2586 (51)	
Hospital outpatient clinics	1140 (27)	355 (18)	58 (7)	2466 (49)	
**Nicotine Dependence level**^b^					<0.001
Light/moderate	2030 (49)	986 (51)	374 (45)	2287 (45)	
Severe	2119 (51)	958 (49)	449 (55)	2765 (55)	
**Smoking years**					
Mean ± SD	22.9 ± 11.9	22 ± 11.9	20.7 ± 10.5	22.8 ± 11.1	<0.001
<10	441 (11)	260 (13)	80 (10)	442 (9)	<0.001
10–19	1071 (26)	494 (25)	281 (34)	1406 (28)	
20–29	1243 (30)	562 (29)	267 (32)	1569 (31)	
30–39	899 (22)	411 (21)	134 (16)	1089 (22)	
≥40	495 (12)	217 (11)	61 (7)	546 (11)	
**Smoking cessation service**					
Clinic visits, mean ± SD	1.6 ± 1.1	1.5 ± 1.0	1.6 ± 0.8	1.8 ± 0.9	<0.001
Once	2573 (62)	1321 (68)	500 (61)	2211 (44)	<0.001
Twice or more	1576 (38)	623 (32)	323 (39)	2841 (56)	
Medication use duration, week					
Mean ± SD	2.4 ± 1.5	2.2 ± 1.4	2.3 ± 1.4	2.9 ± 1.5	<0.001
1	1483 (36)	820 (42)	337 (41)	1223 (24)	<0.001
2	1192 (29)	535 (28)	181 (22)	1113 (22)	
≥3	1474 (36)	589 (30)	305 (37)	2716 (54)	

NRT, nicotine replacement therapy; SD, standard deviation.

^a^ *P*-value for χ^2^ test or one-way ANOVA.

^b^ Nicotine dependence level defined by the Fagerström Test for Nicotine Dependence (FTND) score [19]: 0–3, light; 4–6, moderate; 7–10, severe.

Compared with other treatment groups, varenicline users were more likely to have completed college or higher education, resided in North Taiwan, received smoking cessation services in hospital outpatient clinics, and had severe dependence (FTND score of 7–10) (all *P* < 0.001). Varenicline users also visited smoking cessation services more often and had the longest duration of medication use; 56% visited smoking cessation clinics at least twice (compared to 32% to 39% for NRT or bupropion users, *P* < 0.001) during follow-up, and 54% (compared to 30% to 37% of NRT or bupropion users, *P* < 0.001) received prescription for 3 weeks or longer. Mean smoking years were similar between users of varenicline or NRT, and fewest for bupropion users: 58% of bupropion users compared to 62% to 64% of users of NRT or varenicline had more than 20 years of smoking (*P* < 0.001).

In multivariable regression models (Table 2), point-prevalence of abstinence 6 months later was associated with the following: older age; visiting hospital outpatient clinics; light or moderate nicotine dependence level; fewer smoking years; higher frequency of clinic visits; and longer duration of medication use. Men were no more likely than women to be abstinent after 6 months.

**Table 2:** Multivariable-adjusted OR (95% CI) for 7-day, 1-month or 6-month point-prevalence of abstinence after 6 months in relation to baseline characteristics

	Adjusted OR (95% CI) for point-prevalence of abstinence		
	7-day	1-month	6-month
**Age** (per 10 year change)	1.19 (1.11–1.26)	1.19 (1.12–1.27)	1.19 (1.10–1.29)
**Sex**			
Female	1 (Reference)	1 (Reference)	1 (Reference)
Male	0.96 (0.86–1.08)	0.95 (0.84–1.07)	1.14 (0.97–1.33)
**Education**			
None, elementary, or unknown	1 (Reference)	1 (Reference)	1 (Reference)
Junior high school	0.84 (0.70–1.01)	0.86 (0.72–1.04)	0.95 (0.75–1.21)
Senior high school	0.97 (0.82–1.14)	0.97 (0.82–1.15)	1.12 (0.90–1.39)
College or higher	1.25 (1.05–1.48)	1.27 (1.07–1.50)	1.29 (1.03–1.60)
**Marital status**			
Single	1 (Reference)	1 (Reference)	1 (Reference)
Married	1.18 (1.05–1.31)	1.19 (1.07–1.34)	1.12 (0.97–1.29)
Other	0.85 (0.72–1.00)	0.87 (0.74–1.02)	0.82 (0.66–1.01)
**Geographic area**			
North	1 (Reference)	1 (Reference)	1 (Reference)
West-central	1.08 (0.97–1.20)	1.08 (0.97–1.20)	1.05 (0.92–1.20)
South	1.02 (0.92–1.12)	1.02 (0.92–1.12)	0.96 (0.85–1.09)
**Medical institution**			
Community Clinics	1 (Reference)	1 (Reference)	1 (Reference)
Hospital outpatient clinics	1.18 (1.08–1.29)	1.20 (1.10–1.31)	1.24 (1.11–1.38)
**Nicotine dependence level**^**a**^			
Light/moderate	1 (Reference)	1 (Reference)	1 (Reference)
Severe	0.88 (0.82–0.94)	0.88 (0.82–0.94)	0.91 (0.83–0.98)
**Smoking years** (per 10 years)	0.71 (0.65–0.77)	0.72 (0.66–0.78)	0.81 (0.73–0.90)
**Smoking cessation service**			
Clinic visits	1.23 (1.17–1.30)	1.24 (1.17–1.31)	1.16 (1.09–1.24)
Medication use duration (week)	1.15 (1.11–1.19)	1.15 (1.11–1.19)	1.12 (1.07–1.17)

*aOR*, adjusted odds ratio; *CI*, confidence interval.

^a^ Nicotine dependence level defined by the Fagerström Test for Nicotine Dependence (FTND) score [19]: 0–3, light; 4–6, moderate; 7–10, severe.

### Comparative effectiveness of smoking cessation modalities

**Fig 2:**
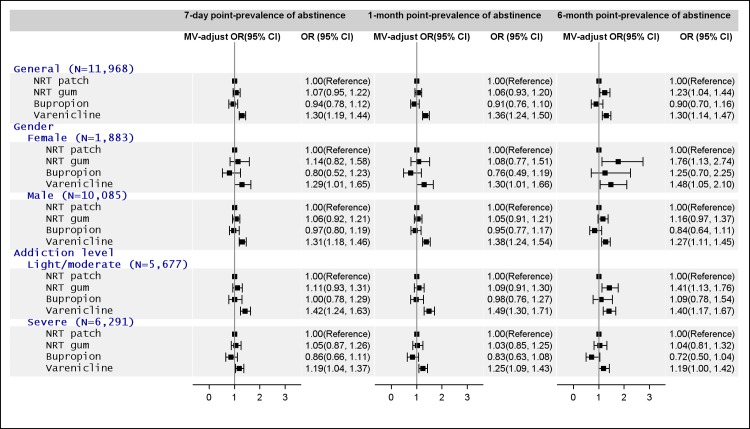
Relative effectiveness of smoking cessation medications for point-prevalence of abstinence after 6 months, with stratifications by sex and nicotine dependence level. ^a^ Models included: age, sex, education, marital status, geographic area, medical institution, nicotine dependence level, smoking years, smoking cessation clinic visit and medication use duration. Sex and nicotine dependence level was not included in analyses stratified by the covariate. *MV-adjust*, multivariable-adjusted; *OR*, odds ratio; *CI*, confidence interval; *NRT*, nicotine replacement therapy.

In telephone interviews, 34% of varenicline users reported their last smoking event to be at least 7 days ago (7-day point-prevalence) and 16% reported it was 180 days ago (6-month point-prevalence); 7-day point-prevalence was 24% in bupropion users, and 26% among users of NRT patch or gum. The 6-month point-prevalence was 10–12% for bupropion or NRT users. Compared to NRT patch users, varenicline users had 30–36% greater odds of self-reporting that the last time they smoked was at least 7 days ago, 1 month ago or 6 months ago, after controlling for age, sex, smoking years, nicotine dependence level, period of medication use, visits to smoking cessation clinics and other socio-economic variables (Fig 2). NRT gum users were more likely than patch users to have self-reported the last time of smoking to be at least 6 months ago. Differential effectiveness of bupropion versus NRT patch on point-prevalence of abstinence was not observed.

### Stratified analyses

The relative effectiveness of the four smoking cessation treatments was similar in male and female smokers (Fig 2). Varenicline supported both males and females in maintaining smoking cessation behavior, with a greater 7-day point-prevalence (male: OR = 1.31, *P* < 0.001; female: OR = 1.29, *P* = 0.043), 1-month point-prevalence (male: OR = 1.38, *P* < 0.001; female: OR = 1.30, *P* = 0.040) and 6-month point-prevalence (male: OR = 1.27, *P* < 0.001; female: OR = 1.48, *P* = 0.027). NRT gum seemed to be more effective than NRT patch among female users based on the 6-month point-prevalence (OR = 1.76, *P* = 0.013). Such an association was not observed in men.

Among those with light/moderate dependence (addiction level), varenicline was more effective than NRT patch on the 7-day (OR = 1.42, *P* <0.001), 1-month (OR = 1.49, *P* <0.001) and 6-month point abstinence (OR = 1.40, *P* < 0.001). Similar results were seen in participants with severe nicotine dependence (Fig 2). The effect size of varenicline relative to NRT patch was consistently higher among smokers with light/moderate nicotine dependence (OR ranged from 1.40 to 1.49) than among smokers with severe nicotine dependence (OR ranged from 1.19 to 1.25).

### Sensitivity analyses

The distribution of baseline characteristics among 14,847 smokers who refused the telephone interview or were lost to follow-up (non-respondents in S1 Table) was similar to participants who failed to remain abstinent from smoking, including the proportion of participants who had severe nicotine dependence, used smoking cessation services once and received medications for less than 2 weeks. In addition, non-respondents were more likely to have used an NRT patch or NRT gum than varenicline (56.9% vs 36.3% in non-respondents; 53.2% vs 39.3% in respondents who failed to remain abstinence). Conversely, almost half of respondents who remained abstinent used varenicline (49.8%). We therefore considered non-respondents as failures in smoking cessation in the sensitivity analyses. Results of sensitivity analyses were similar to those of the analytic sample (S2 and S3 Tables). Two alternative definitions of smoking cessation outcomes were also tested: 1) based on the telephone interview results regardless of medication switching/add-on in the next treatment course after the first 90-days; and 2) in addition to switch/add on, we also defined continuing the same medication in the second treatment course as failure in smoking cessation. These sensitivity analyses using different outcome definitions consistently showed greater effectiveness of varenicline relative to NRT patch.

## Discussion

This population-based cohort study constituted a unique investigation that has directly compared common pharmacologic approaches to smoking cessation using large-scale prospective data from an Asian population in a real-world setting. Specifically, this study used national data from Taiwan to compare the effect of four smoking cessation modalities, NRT patch, NRT gum, bupropion and varenicline, on rates of smoking cessation after 6 months. The 6-month cessation rates were highest among varenicline users (16%) and lowest among bupropion users (10%). Varenicline also appeared to perform better than NRT patch in achieving smoking cessation, irrespective of users’ sex or nicotine dependence level. To our knowledge, this is the first prospective cohort study in clinical practice with a large sample size reporting the comparative effectiveness of smoking cessation medications in women and smokers with light/moderate nicotine dependence.

Pharmacologic intervention improves the likelihood of smoking cessation at 6 months or more, compared to no intervention, as reported by the US Preventive Services Task Force [3]. The EAGLES study was the first head-to-head clinical trial and provided direct evidence that varenicline was superior to bupropion, nicotine patch or placebo in achieving carbon monoxide-confirmed continuous abstinence rates for weeks 9–24 in smokers who received 12-week treatment [11]. The abstinence rate in the EAGLES study was 21.8%, 16.2%, 15.7%, and 9.4% for those who received varenicline, bupropion, nicotine patch or placebo, respectively. In network meta-analysis with direct or indirect comparison of clinical trials, varenicline provided 50–70% greater odds of smoking cessation than NRT (gum or patch) or bupropion alone [4, 5, 21], similar to findings in the EAGLES study [11]. In the few comparative studies of different smoking cessation medications in community settings, smokers who used varenicline self-reported 1.6- to 3.8-times greater abstinence rates after 6 months than those who used an NRT patch [8] or any NRT prescription [10]. Our study cohort drawn from smoking cessation clinics throughout Taiwan affirms that smokers at their first attempt to quit respond better to varenicline than to an NRT patch; it also corroborates findings from the EAGLES study or meta-analyses that bupropion is not superior to NRT patch in smoking cessation at 6 months [5, 11]. As such, our results constitute direct evidence that the relative effectiveness of smoking cessation medications seen in clinical trials extrapolates to a ‘real-world’ population of diverse individuals in Asia.

Women in clinical trials had greater difficulty quitting smoking than men when randomly assigned to placebo [13, 20] (13, 19), NRT patch [16] or bupropion [15]; however, varenicline was more effective in women than in men for short- and immediate-term abstinence [20], although this advantage disappeared after 1 year. In observational studies, self-reported rates of quitting smoking and the successful smoking cessation rates were similar among older men and women, but higher in women than in men among adults younger than 50 [22]. Among smokers who received NRT in one clinic, women were less likely than men to be abstinent after 1 year [12]. Conversely, our study did not find differential abstinence rates between the sexes, regardless of which smoking cessation medications they had used. Furthermore, varenicline had comparable magnitudes of association in women and men relative to NRT patch. Findings in women versus men cannot be compared directly between these studies due to their distinct designs, populations, countries, and objectives. Studies in other clinical settings are warranted to confirm our findings and to evaluate the responses of middle-aged and older women who receive varenicline. Of note, in stratified analyses of women, OR point estimate for 6-month point-prevalence of abstinence was greatest in NRT patch users. However, OR and interval estimates suggested a null association for 7-day and 1-month point-prevalence and a positive association for 6-month point-prevalence comparing NRT gum users to NRT patch users. These inconsistent findings need cautious interpretation.

From 2005 to 2011, the prevalence of tobacco smoking remained stable but the proportion of heavy smokers declined significantly in the US [17]. Research on smoking cessation medications and clinical practice has mainly focused on heavy smokers or those with high nicotine dependence [11]; studies on light smokers are sparse. Although light smokers were more likely to try to quit smoking, they were less likely to use smoking cessation medications [23]; indeed, varenicline was more likely to be prescribed to heavy smokers than light smokers [24]. Light smokers who failed to quit smoking were also more likely to receive varenicline than NRT [25]. Yet, in a comparative clinical trial among moderate nicotine-dependents who smoked less than 20 cigarettes per day, varenicline did not show superior effectiveness to NRT [18]. Congruent with previous studies [24, 25], heavily nicotine-dependent smokers were more likely to receive varenicline than NRT in the current study. In contrast to clinical trial findings [18], new quitters in our study with light/moderate nicotine dependence responded better to varenicline than NRT patch, as well as smokers strongly addicted to nicotine, to a lesser degree. Further investigation assessing efficacy stratified by dependence severity is needed.

While our study included a very large sample size and presented findings in clinical practice, this study had several limitations. First, smoking abstinence was self-reported. Over-reporting of tobacco abstinence would be expected to be universal for each comparison group and thus rendered a non-differential misclassification in outcomes–ratio estimates would likely be biased toward the null and under-estimated. Second, we were unable to control for unobserved confounding factors, such as confounding by indication (for example, physicians may prescribe varenicline to smokers who were highly motivated to quit or those with severe nicotine dependence). We could not collect information about whether patients used other methods besides smoking cessation medications; these circumstances would bias the estimates. We performed multivariable regression modellings and stratified analyses to control for known confounding factors. Residual confounding may still exist and we cannot avoid chance findings from multiple comparisons in stratified analyses. Third, there was a high non-response rate at the follow-up phone interview. In sensitivity analyses, we considered non-respondents failures in smoking cessation and found similar comparative effectiveness to the original study design. Fourth, as many studies used medical records or claim datasets, we cannot confirm whether smokers actually used the prescribed medication. Participants may not have adhered to the treatment during follow-up. Medication-related adverse events may reduce adherence and thus abstinence rate. The discontinuation rate due to medication-related adverse events, however, was 1–5% in large-scale clinical trials [11, 18, 26] and should not significantly affect our results. Additionally, treating non-respondents as failures likely accounted for this. Results from the sensitivity analyses agreed with findings in the original analytic sample. Fifth, we lacked data on important variables such as smoking status of family members, which may have affected smoking cessation; however, these factors were less likely to influence quitters to select particular modalities, and may not substantially alter the efficacy of varenicline versus NRT patch. Lastly, our study results from one single-payer health care system in an Asian population may not extrapolate to different health care systems or non-Asian populations.

Clinical trials have shown smoking cessation medications to be more effective than placebo for achieving abstinence and suggested that varenicline may offer greater effectiveness than bupropion and NRT. In this large, national sample of smokers who received mono-pharmacotherapy for their first attempt to quit smoking, varenicline users had a greater likelihood of self-reporting abstinence after 6 months than users of NRT patch or bupropion. Female smokers and those with light/moderate nicotine dependence–subgroups that usually have a high failure rate in quitting smoking–also responded better to varenicline than NRT patch. These findings not only provide direct evidence in a real-world setting and complement results from clinical trials, but also provide policy makers, payers, and health care providers with valuable information about prescribing and reimbursing medication. Future studies should investigate the long-term effect or the effect on preventing relapse, analyze cost-effectiveness among different smoking populations, and assess the impacts of comorbid conditions, medical history, or the strong motivation to quit.

## Supporting Information

S1 Table Baseline characteristics between those who did and did not respond to phone interview after 6 months (DOCX)

S2 Table Sensitivity analyses for multivariable-adjusted odds ratio (OR) and 95% confidence interval (CI) for 7-day, 1-month and 6-month point-prevalences by smoking cessation medications including 11,968 respondents and 14,847 non-respondents who were considered as failures in smoking cessation (DOCX)

S3 Table Sensitivity analyses for stratified analyses by sex and separately by nicotine dependence level, among 11,968 respondents and 14,847 non-respondents who were treated as failures in smoking cessation. (DOCX)
