# Supplementary material for: Comparative Effectiveness of Smoking Cessation Medications: A National Prospective Cohort From Taiwan
Source: PLoS One. 2016 Nov 28;11(11):e0166992. doi: 10.1371/journal.pone.0166992 (PMC5125644; doi:10.1371/journal.pone.0166992)
Supplement: S1 Table — (DOCX) [file pone.0166992.s001.docx]

| **S1 Table.** Baseline characteristics between those who did and did not respond to phone interview after 6 months | | | | | | | | | | | |
| --- | --- | --- | --- | --- | --- | --- | --- | --- | --- | --- | --- |
|  | **Respondents** | | | | | | | |  | **^*^Non-respondents**  **(n=14,847)** | |
|  | **Total**  **(n=11,968)** | |  | **Abstinence from smoking**  **(n=3,294)** | |  | **Fail to remain abstinent (n=8,674)** | |  |  |  |
|  | n | % |  | n | % |  | n | % |  | n | % |
| **Medication** |  |  |  |  |  |  |  |  |  |  |  |
| NRT patch | 4,149 | 34.7 |  | 1,005 | 30.5 |  | 3,144 | 36.3 |  | 5,635 | 38.0 |
| NRT gum | 1,944 | 16.2 |  | 475 | 14.4 |  | 1,469 | 16.9 |  | 2,808 | 18.9 |
| Bupropion | 823 | 6.9 |  | 175 | 5.3 |  | 648 | 7.5 |  | 1,009 | 6.8 |
| Varenicline | 5,052 | 42.2 |  | 1,639 | 49.8 |  | 3,413 | 39.3 |  | 5,395 | 36.3 |
| **Sociodemographic characteristics** | | |  |  |  |  |  |  |  |  |  |
| **Age, year** |  |  |  |  |  |  |  |  |  |  |  |
| Mean (SD) | 43.7 | (12.6) |  | 44.5 | (12.9) |  | 43.4 | (12.5) |  | 44.3 | (14.0) |
| 18-24 | 622 | 5.2 |  | 146 | 4.4 |  | 476 | 5.5 |  | 973 | 6.5 |
| 25-34 | 2,734 | 22.8 |  | 729 | 22.1 |  | 2,005 | 23.1 |  | 3,415 | 23.0 |
| 35-44 | 3,436 | 28.7 |  | 902 | 27.4 |  | 2,534 | 29.2 |  | 3,785 | 25.5 |
| 45-54 | 2,893 | 24.2 |  | 825 | 25.1 |  | 2,068 | 23.8 |  | 3,341 | 22.5 |
| 55-64 | 1,598 | 13.4 |  | 470 | 14.3 |  | 1,128 | 13.0 |  | 2,058 | 13.9 |
| 65+ | 685 | 5.7 |  | 222 | 6.7 |  | 463 | 5.3 |  | 1,275 | 8.6 |
| **Gender** |  |  |  |  |  |  |  |  |  |  |  |
| Female | 1,883 | 15.7 |  | 503 | 15.3 |  | 1,380 | 15.9 |  | 2,826 | 19.0 |
| Male | 10,085 | 84.3 |  | 2,791 | 84.7 |  | 7,294 | 84.1 |  | 12,021 | 81.0 |
| **Geographic area** |  |  |  |  |  |  |  |  |  |  |  |
| North | 5,768 | 48.2 |  | 1,592 | 48.3 |  | 4,176 | 48.1 |  | 6,645 | 44.8 |
| West-central | 2,771 | 23.2 |  | 767 | 23.3 |  | 2,004 | 23.1 |  | 3,592 | 24.1 |
| South | 3,429 | 28.7 |  | 935 | 28.4 |  | 2,494 | 28.8 |  | 4,610 | 31.1 |
| **Medical institution** |  |  |  |  |  |  |  |  |  |  |  |
| Clinics | 7,949 | 66.4 |  | 2,034 | 61.8 |  | 5,915 | 68.2 |  | 9,874 | 66.5 |
| Hospital | 4,019 | 33.6 |  | 1,260 | 38.3 |  | 2,759 | 31.8 |  | 4,973 | 33.5 |
| **Nicotine dependence level** | |  |  |  |  |  |  |  |  |  |  |
| Light/moderate | 5,677 | 47.4 |  | 1,769 | 53.7 |  | 3,908 | 45.0 |  | 7,027 | 47.3 |
| Severe | 6,291 | 52.6 |  | 1,525 | 46.3 |  | 4,766 | 55.0 |  | 7,820 | 52.7 |
| **Smoking years** |  |  |  |  |  |  |  |  |  |  |  |
| Mean (SD) | 22.5 | (11.5) |  | 22.7 | (11.6) |  | 22.5 | (11.4) |  | 22.5 | (12.5) |
| <10 | 1,223 | 10.2 |  | 330 | 10.0 |  | 893 | 10.3 |  | 1,772 | 11.9 |
| 10-19 | 3,252 | 27.2 |  | 897 | 27.2 |  | 2,355 | 27.2 |  | 4,112 | 27.7 |
| 20-29 | 3,641 | 30.4 |  | 961 | 29.2 |  | 2,680 | 30.9 |  | 4,188 | 28.2 |
| 30-39 | 2,533 | 21.2 |  | 734 | 22.3 |  | 1,799 | 20.7 |  | 2,796 | 18.8 |
| ≥40 | 1,319 | 11.0 |  | 372 | 11.3 |  | 947 | 10.9 |  | 1,979 | 13.3 |
| **Smoking cessation service** | | |  |  |  |  |  |  |  |  |  |
| Clinic visits |  |  |  |  |  |  |  |  |  |  |  |
| Mean (SD) | 1.7 | (1.0) |  | 1.9 | (1.1) |  | 1.6 | (0.9) |  | 1.5 | (1.0) |
| Once | 6,605 | 55.2 |  | 1,350 | 41.0 |  | 5,255 | 60.6 |  | 9629 | 64.9 |
| Twice or more | 5,363 | 44.8 |  | 1,944 | 59.0 |  | 3419 | 39.4 |  | 5218 | 35.1 |
| Medication use duration, weeks | | |  |  |  |  |  |  |  |  |  |
| Mean (SD) | 2.5 | (1.5) |  | 2.9 | (1.6) |  | 2.4 | (1.5) |  | 2.3 | (1.5) |
| 1 week | 3,863 | 32.3 |  | 734 | 22.3 |  | 3129 | 36.1 |  | 5,857 | 39.4 |
| 2 weeks | 3,021 | 25.2 |  | 711 | 21.6 |  | 2310 | 26.6 |  | 3,900 | 26.3 |
| ≥3 weeks | 5,084 | 42.5 |  | 1,849 | 56.1 |  | 3235 | 37.3 |  | 5,090 | 34.3 |
| * Non-respondent included 14,812 participants who refused interview or uncontactable and 35 participants with unknown smoking status in the phone interview. | | | | | | | | | | | |
