# Supplementary material for: Comparative Effectiveness of Smoking Cessation Medications: A National Prospective Cohort From Taiwan
Source: PLoS One. 2016 Nov 28;11(11):e0166992. doi: 10.1371/journal.pone.0166992 (PMC5125644; doi:10.1371/journal.pone.0166992)
Supplement: S2 Table — (DOCX) [file pone.0166992.s002.docx]

| **S2 Table.** Sensitivity analyses for multivariable-adjusted odds ratio (OR) and 95% confidence interval (CI) for 7-day, 1-month and 6-month point-prevalences by smoking cessation medications including 11,968 respondents and 14,847 non-respondents who were considered as failures in smoking cessation | | | | | | | | | | | |
| --- | --- | --- | --- | --- | --- | --- | --- | --- | --- | --- | --- |
|  | **7-day point-prevalence** | | |  | **1-month point-prevalence** | | |  | **6-month point-prevalence** | | |
|  | OR | (95% CI) | |  | OR | (95% CI) | |  | OR | (95% CI) | |
| **Smoking cessation medication** | | | | | | | | | | | |
| NRT patch | (Reference) | | |  | (Reference) | | |  | (Reference) | | |
| NRT gum | 1.05 | (0.93-1.19) | |  | 1.03 | (0.90-1.17) | |  | 1.19 | (1.01-1.40) | |
| Bupropion | 0.90 | (0.75-1.08) | |  | 0.87 | (0.73-1.05) | |  | 0.87 | (0.68-1.11) | |
| Varenicline | 1.33 | (1.21-1.46) | |  | 1.34 | (1.22-1.48) | |  | 1.28 | (1.13-1.45) | |
| **Age** (40 y/o ±10 years) | 1.40 | (1.32-1.49) | |  | 1.40 | (1.31-1.49) | |  | 1.35 | (1.25-1.47) | |
| **Gender** |  |  |  |  |  |  |  |  |  |  |  |
| Female | (Reference) | | |  | (Reference) | | |  | (Reference) | | |
| Male | 0.80 | (0.71-0.89) | |  | 0.79 | (0.71-0.89) | |  | 0.99 | (0.84-1.16) | |
| **Education** |  |  |  |  |  |  |  |  |  |  |  |
| Elementary, no education, or unknown | (Reference) | | |  | (Reference) | | |  | (Reference) | | |
| Junior high school | 5.14 | (4.24-6.24) | |  | 5.26 | (4.32-6.42) | |  | 4.68 | (3.58-6.12) | |
| Senior high school | 6.28 | (5.29-7.45) | |  | 6.19 | (5.19-7.38) | |  | 5.73 | (4.50-7.29) | |
| College or above | 8.14 | (6.83-9.69) | |  | 8.10 | (6.78-9.69) | |  | 6.59 | (5.16-8.42) | |
| **Marital status** |  |  |  |  |  |  |  |  |  |  |  |
| Single | (Reference) | | |  | (Reference) | | |  | (Reference) | | |
| Married | 1.04 | (0.93-1.16) | |  | 1.06 | (0.95-1.18) | |  | 1.02 | (0.88-1.17) | |
| Other | 0.21 | (0.18-0.25) | |  | 0.22 | (0.18-0.26) | |  | 0.23 | (0.18-0.30) | |
| **Geographic area** |  |  |  |  |  |  |  |  |  |  |  |
| North | (Reference) | | |  | (Reference) | | |  | (Reference) | | |
| West-central | 1.19 | (1.07-1.32) | |  | 1.18 | (1.06-1.31) | |  | 1.12 | (0.98-1.28) | |
| South | 1.08 | (0.98-1.19) | |  | 1.08 | (0.98-1.19) | |  | 1.00 | (0.88-1.13) | |
| **Medical institution** |  |  |  |  |  |  |  |  |  |  |  |
| Clinics | (Reference) | | |  | (Reference) | | |  | (Reference) | | |
| Hospitals | 1.05 | (0.96-1.15) | |  | 1.07 | (0.97-1.17) | |  | 1.12 | (1.00-1.26) | |
| **Nicotine dependence level** |  |  |  |  |  |  |  |  |  |  |  |
| Light or moderate | (Reference) | | |  | (Reference) | | |  | (Reference) | | |
| Severe | 0.72 | (0.67-0.79) | |  | 0.73 | (0.67-0.79) | |  | 0.83 | (0.74-0.92) | |
| **Smoking years** (40 years ±10 years) | 0.91 | (0.85-0.97) | |  | 0.90 | (0.84-0.96) | |  | 0.93 | (0.85-1.01) | |
| **Smoking cessation service** |  |  |  |  |  |  |  |  |  |  |  |
| Smoking cessation clinic visits | 1.18 | (1.12-1.24) | |  | 1.18 | (1.12-1.25) | |  | 1.14 | (1.07-1.21) | |
| Medication use period, weeks | 1.14 | (1.10-1.18) | |  | 1.13 | (1.09-1.17) | |  | 1.11 | (1.06-1.16) | |
| Models included all covariates shown in the S2 Table. | | | | | | | | | | | |
