# Supplementary material for: Comparative Effectiveness of Smoking Cessation Medications: A National Prospective Cohort From Taiwan
Source: PLoS One. 2016 Nov 28;11(11):e0166992. doi: 10.1371/journal.pone.0166992 (PMC5125644; doi:10.1371/journal.pone.0166992)
Supplement: S3 Table — (DOCX) [file pone.0166992.s003.docx]

| **S3 Table.** Sensitivity analyses for stratified analyses by sex and separately by nicotine dependence level, among 11,968 respondents and 14,847 non-respondents who were treated as failures in smoking cessation. | | | | | | | | | | | |
| --- | --- | --- | --- | --- | --- | --- | --- | --- | --- | --- | --- |
|  | **7-day point-prevalence** | | |  | **1-month point-prevalence** | | |  | **6-month point-prevalence** | | |
|  | ^1^OR | (95% CI) | |  | ^1^OR | (95% CI) | |  | ^1^OR | (95% CI) | |
| **Stratified analysis by dependence level** | | | |  |  |  |  |  |  |  |  |
| **Light/moderate dependence** | |  |  |  |  |  |  |  |  |  |  |
| NRT patch | (Reference) | | |  | (Reference) | | |  | (Reference) | | |
| NRT gum | 1.07 | (0.90-1.27) | |  | 1.04 | (0.87-1.24) | |  | 1.37 | (1.10-1.70) | |
| Bupropion | 0.96 | (0.75-1.23) | |  | 0.93 | (0.72-1.20) | |  | 1.04 | (0.74-1.46) | |
| Varenicline | 1.42 | (1.24-1.63) | |  | 1.45 | (1.26-1.66) | |  | 1.37 | (1.15-1.64) | |
| **Severe dependence** |  |  |  |  |  |  |  |  |  |  |  |
| NRT patch | (Reference) | | |  | (Reference) | | |  | (Reference) | | |
| NRT gum | 1.04 | (0.86-1.25) | |  | 1.01 | (0.84-1.22) | |  | 1.01 | (0.80-1.29) | |
| Bupropion | 0.83 | (0.64-1.08) | |  | 0.81 | (0.62-1.06) | |  | 0.71 | (0.49-1.02) | |
| Varenicline | 1.24 | (1.08-1.42) | |  | 1.24 | (1.08-1.43) | |  | 1.18 | (0.99-1.41) | |
| **Stratified analysis by sex** |  |  |  |  |  |  |  |  |  |  |  |
| **Female** |  |  |  |  |  |  |  |  |  |  |  |
| NRT patch | (Reference) | | |  | (Reference) | | |  | (Reference) | | |
| NRT gum | 1.07 | (0.77-1.48) | |  | 1.03 | (0.74-1.44) | |  | 1.70 | (1.09-2.64) | |
| Bupropion | 0.75 | (0.49-1.15) | |  | 0.72 | (0.46-1.11) | |  | 1.19 | (0.66-2.12) | |
| Varenicline | 1.21 | (0.95-1.54) | |  | 1.22 | (0.96-1.55) | |  | 1.43 | (1.01-2.02) | |
| **Male** |  |  |  |  |  |  |  |  |  |  |  |
| NRT patch | (Reference) | | |  | (Reference) | | |  | (Reference) | | |
| NRT gum | 1.05 | (0.91-1.20) | |  | 1.02 | (0.89-1.17) | |  | 1.12 | (0.94-1.34) | |
| Bupropion | 0.94 | (0.77-1.15) | |  | 0.91 | (0.75-1.12) | |  | 0.81 | (0.61-1.07) | |
| Varenicline | 1.35 | (1.22-1.50) | |  | 1.37 | (1.23-1.52) | |  | 1.25 | (1.10-1.43) | |
| ^1^Modesls included age, sex (except for stratified analyses by sex), education, marital status, geographic region, smoke-years, nicotine-dependence level (except for stratified analyses by dependence severity), medical institution, number of clinic visits, and medication duration. | | | | | | | | | | | |
